# Effects of Chlortetracycline Rumen-Protected Granules on Rumen Microorganisms and Its Diarrhea Therapeutic Effect

**DOI:** 10.3389/fvets.2022.840442

**Published:** 2022-02-18

**Authors:** Yang Yu, Xin Li, Ziyao Liu, Ying Xu, Yue Shen, Guoji Li, Xianhui Huang

**Affiliations:** ^1^Guangdong Key Laboratory for Veterinary Drug Development and Safety Evaluation, College of Veterinary Medicine, South China Agricultural University, Guangzhou, China; ^2^Guangdong Laboratory for Lingnan Modern Agriculture, South China Agricultural University, Guangzhou, China

**Keywords:** chlortetracycline, rumen fluid, qPCR, 16S rRNA, diarrhea, lamb

## Abstract

Chlortetracycline is a broad-spectrum antibiotic used as an oral medication in ruminants. However, this antibiotic affects the rumen microbial population, thereby upsetting the normal microbiota of ruminants. This study determined whether our newly developed chlortetracycline rumen-protected granules are relatively harmless to rumen microorganisms while effective against lamb *E. coli* diarrhea. We used a qPCR assay to quantify selected rumen microorganisms from lambs treated with or without oral chlortetracycline. We also assessed bacterial diversity in the rumen by 16S rRNA gene sequencing. Lambs were divided into three groups: one group given with oral chlortetracycline granules for 7 days; one group with chlortetracycline premix; and one without treatment. Rumen fluid was collected on 0 d, 7 d, and 14 d of the experiment. In the therapeutic effect trial, cases of naturally *E. coli*-infected lamb with diarrhea were selected and divided into low, medium, and high dose groups of granules, premix, infection control, and healthy control groups. Treatments were continuously administered for 7 days, and animals were observed for 14 days after drug withdrawal to score and evaluate the treatment effect. Results of qPCR and 16S rRNA gene sequencing showed that the granules could diminish the impact of chlortetracycline on rumen microorganisms compared with the premix. The diarrhea therapeutic effect trial showed that the oral administration of the chlortetracycline rumen-protected granules at the dose of 30 mg/kg·bw/d for 7 days could effectively treat lamb diarrhea caused by *E. coli*. In conclusion, we provide a new drug preparation of chlortetracycline that can diminish the effect on the rumen microbiota while treating diarrhea caused by *E. coli*.

## Introduction

The rumen is a unique digestive organ that provides a complex microbial ecosystem for ruminants. Through microbial fermentation, biomass that cannot be digested enzymatically by the ruminant is degraded and converted into volatile fatty acids (VFA), fermentation gas, and heat, promoting body growth and development (1). In the rumen microbial ecosystem, bacteria have the most significant number and contribute the most during microbial digestion. Moreover, the development of the rumen plays a vital role in the growth of young ruminants and, in turn, directly affects the ability of adults to digest nutrients. Hence, it is crucial to identify which factors influence the development of rumen microorganisms in young ruminants. Common factors affecting rumen microorganisms include diet (2–5), environment (6), feeding methods (7, 8), and drugs (9, 10).

Bacterial diarrhea caused by *Escherichia coli (E. coli)* is widely prevalent in calves and lambs, causing a high mortality rate that results in significant losses to the ruminant breeding industry. Chlortetracycline (CTC) is a broad-spectrum antibiotic that the FDA has approved to treat diarrhea in calves caused by *E. coli*. However, there are studies on the growth-promoting effect of chlortetracycline for lambs, with no sufficient data on its treatment efficacy against *E. coli* diarrhea. In addition, due to ruminants' unique digestive physiological structure, rumen microbial fermentation during oral administration may degrade antibiotics, thus reducing the efficacy. More importantly, antibiotics can inhibit or kill the rumen microbial community, affecting the fermentation and degradation of feed in the rumen (11–13). Furthermore, oral administration of chlortetracycline premix leads to the early release of the drug in the rumen due to its immediate dissolution effect, resulting in a series of adverse reactions, such as non-rumination, rumen swelling, acidosis, and digestive disorders. Given the current findings that feeding chlortetracycline can change the rumen microbial population (14), we independently developed chlortetracycline rumen-protected granules to reduce the release of drugs in the rumen, thereby diminishing the effect of oral chlortetracycline on rumen microorganisms.

Therefore, to better evaluate the self-developed chlortetracycline rumen-protected granules, the chlortetracycline premix was selected as the control drug. Furthermore, eight representative rumen microorganisms were selected for quantitative detection by qPCR and 16S rRNA gene sequencing to test whether the granule can diminish the number of rumen microorganisms compared to the premix. In addition, we also carried out a trial against *E. coli* diarrhea in lambs to test whether the prepared granules have a good diarrhea treatment effect and select the preliminarily effective dose.

## Materials and Methods

### Animals and Experimental Design

#### Animal Grouping for qPCR and 16s rRNA Gene Sequencing

Eighteen two-month-old weaned lambs, 9 males and 9 females (13.86 ± 1.48 kg), were used in a 2 week experiment. Lambs were first acclimatized for 1 week in the experimental station. After 1 week of acclimatization, lambs were randomly divided into 3 groups (*n* = 6 for each group). Lambs in group I were fed with concentrate and forage (without chlortetracycline) as the control group. Group II was provided with the same feed and then given with chlortetracycline rumen-protected granules orally at a dose of 30 mg/kg body weight (b.w.) for 7 days. Group III was provided with 10% chlortetracycline premix orally at a dose of 30 mg/kg body weight (b.w.) for 7 days.

#### Animal Grouping for Diarrhea Therapeutic Effect Trial

This experiment selected diarrhea cases of lambs aged 2–3 months naturally infected with *E. coli*. First, the clinical symptoms were examined. A total of 195 diarrhea lambs were initially screened out; then, cotton swabs were collected immediately. Animals were numbered according to the time they were selected. Using the SAS Proc plan program and according to block randomization, the experimental animals were randomly divided into five groups (*n* = 39 for each group): Group I was the infection control group, Group II–IV were the low (15 mg/kg·bw/d), medium (30 mg/kg·bw/d), and high (60 mg/kg·bw/d) dose groups of chlortetracycline rumen-protected granules, respectively, and Group V was the chlortetracycline premix group (30 mg/kg·bw/d). In addition, 39 healthy lambs aged 2–3 months were selected for the healthy control group (VI).

The enrolment criteria included clinically diagnosed with diarrhea symptoms and identified as diarrhea caused by *E. coli* after pathogen identification. Animals with only clinical diarrhea but not identified as *E. coli* were excluded in the statistical analysis. Similarly, animals with other diseases were excluded.

### Rumen Content Sampling

Rumen fluid samples were collected by a stomach tube after feeding in the morning on days 1, 7, and 14 of the trial. To reduce saliva contamination and ensure the accuracy of follow-up test results, we discarded the initially collected 10 mL rumen fluid. Then the rumen fluid was quickly filtered by four layers of gauze and dispensed into centrifuge tubes. All samples were snapped frozen in liquid nitrogen and stored at −80°C.

### DNA Extraction

Frozen samples of rumen fluid were thawed on ice, and then the DNA was extracted from samples using a modified cetyltrimethylammonium bromide (CTAB) extraction protocol. To further optimize the extraction process, after the CTAB water bath heating lysis, we collected the supernatant, added 5 μL RNase A to the supernatant, and incubated it at 37°C for 30 min to remove contaminating RNAs. The other methods are consistent with those described in the literature (15).

### Primers

For *Prevotella species* and *B. fibrisolvens*, the 16S rDNA sequences of different strains of *Prevotella species* and *B. fibrisolvens* were downloaded from GenBank (ncbi.nlm.nih.gov/genbank/). The sequence alignment was carried out using Megalign in DNAStar to find the conserved regions within the species and the specific sequences outside the species. Then, a primer express was used for primer design. Primers designed for the target bacteria are shown in Table 1. For other microorganisms, the primer sequences used are listed in Table 1.

**Table 1:** PCR primers for PCR and real-time PCR assay.

**Target species**	**Primer sequencing**	**Amplicon (bp)**	**Tm (**°**C)**	**References**
*Total bacteria*	F: 5′-CGGCAACGAGCGCAACCC-3′ R: 5′-CCATTGTAGCACGTGTGTAGCC-3′	130	60	(16)
*Prevotella species*	F: 5′- AGACACGGTCCAAACTCCTACGG-3′ R: 5′- CCTCACGCTACTTGGCTGGTTC-3′	85	55	This study
*Butyrivibrio fibrisolvens*	F: 5′- GCGTCTGATTAGCCAGTTGGTGAG-3′ R: 5′- CCCACTGCTGCCTCCCGTAG-3′	126	57	This study
*Ruminococcus flavefaciens*	F: 5′-TCTGGAAACGGATGGTA-3′ R: 5′-CCTTTAAGACAGGAGTTTACAA-3′	295	55	(17)
*Fibrobacter succinogenes*	F: 5′-GTTCGGAATTACTGGGCGTAAA-3′ R: 5′-CGCCTGCCCCTGAACTATC-3′	121	60	(16)
*Ruminococcus albus*	F: 5′-CCCTAAAAGCAGTCTTAGTTCG-3′ R: 5′-CCTCCTTGCGGTTAGAACA-3′	175	55	(17)
*Methanogens*	F: 5′- CCGGAGATGGAACCTGAGAC-3′ R: 5′- CGGTCTTGCCCAGCTCTTATTC-3′	160	57	(18)
*General anaerobic fungi*	F: 5′-GAGGAAGTAAAAGTCGTAACAAGGTTTC-3′ R: 5′-CAAATTCACAAAGGGTAGGATGATT-3′	120	60	(16)

### Conventional PCR and Preparation of Standard Plasmid

The extracted DNA was used as a template for the PCR amplification. The primer sets used for PCR are described in Table 1. The PCR mix contains 2 μL of template, 1 μL of each primer, 12.5 μL of Taq polymerase, and 8.5 μL of sterilized water. PCR cyclic conditions were 95°C for 5 min initial denaturation, then 40 cycles of denaturation at 95°C for 30 s, annealing at 55–60°C for 40 s and extension at 72°C for 40 s, with a final extension at 72°C for 5 min. The size of the PCR product was confirmed by carrying out gel-electrophoresis on 1% agarose gel.

After confirming the exact band locations, the PCR products were excised from the gel and purified using an agarose gel DNA extraction kit (TaKaRa, Japan). The purified PCR products were cloned into a PMD-19T cloning vector. Subsequently, the clones were transformed into DH5-α-competent cells and added into LB liquid medium. The DH5-α-competent cells were cultured at 16°C for 1 h, in which bacterial solution was coated on LB agar plates containing IPTG, X-Gal, and ampicillin. After the blue-white screening, white colonies were selected. The selected colonies were inoculated into the LB liquid medium 100 mg/mL ampicillin and incubated overnight at 37°C while shaking at 160 rpm. The plasmid DNA was extracted from bacteria using a plasmid purification kit (TaKaRa, Japan), according to the manufacturer's instructions. The resulting products were subjected to sequencing, and the sequencing results were compared with Blast on GenBank (ncbi.nlm.nih.gov/genbank/).

### Standard Curve Preparation and qPCR

The number of bacteria in rumen fluid samples was detected and quantified using the absolute standard curve constructed from the cloned plasmids. A 10-fold serial dilution of the plasmid was prepared for the standard curve, with the dilution concentration gradient not <5. Before the qPCR absolute quantification, all tested samples were diluted to 10 ng/μL. The primer sets used for the qPCR are described in Table 1.

The qPCR reaction was performed using the CFX96 real-time detection system (Bio-Rad Laboratories, Inc.). The total volume of each reaction system was 20 μL, including 10 μL of SYBR qPCR Master Mix (Vazyme, Nanjing, China), 2 μL of DNA template, 0.4 μL of each primer, and 7.2 μL of sterile water. The qPCR amplification protocol was as follows: 30 s at 94°C for initial denaturation, then 40 cycles of 10 s at 94°C for denaturing and 30 s at 60°C for annealing and extension. Sterile water replaced the DNA template as no template control (NTC). To avoid the error caused by the operation and ensure the accuracy of the experimental results, all samples were detected with three technical replicates.

### 16S rRNA Gene Sequencing

Genomic DNA was extracted using E.Z.N.A.^®^ Soil DNA Kit. The DNA concentration and purity were determined using NanoDrop 2000, while the DNA integrity was assessed using 1% agarose gel electrophoresis. The V3–V4 hypervariable regions of 16S rRNA were PCR amplified from the microbial genomic DNA using the universal primers: V338F, 5′-ACTCCTACGGGAGGCAGCAG-3′; V806R, 5′- GGACTACHVGGGTWTCTAAT-3′. The PCR has been carried out in triplicate in a 20-μL reaction containing 0.8 μM of each primer, 10 ng template DNA, 4 μL 5× FastPfu Buffer, 2 μL 2.5 mM dNTPs, and 0.4 μL FastPfu Polymerase. The PCR cycle condition was as follows: 3 min initial denaturation at 95°C; 27 cycles of denaturation at 95°C for 30 s, annealing at 55°C for 30 s, and elongation at 72°C for 45 s; and a final extension at 72°C for 10 min. The PCR products were extracted from 2% agarose gels and purified using the Axy Prep DNA Gel Extraction Kit (Axygen Biosciences, USA). Quantitative detection was performed using QuantiFluor™-ST. The sequencing was performed using Illumina MiSeq platform (Illumina, San Diego, USA) 300 × 2 paired-end chemistry. The DNA Sequencing was performed by the Miseq PE300 platform (Majorbio, Shanghai, China).

### Evaluation of Diarrhea Therapeutic Effect

#### Dose of Each Group

Referring to the FDA-approved chlortetracycline for treating bacterial enteritis caused by *E. coli* in calves, the dosage used was 10 milligrams per pound of body weight per day (about 22 mg/ kg·bw/d). To consider the loss of drugs in the rumen and to make the drug concentration in the intestine sufficient, the medium dose of this test was set at 30 mg/kg·bw/d, the low dose was set at 15 mg/ kg·bw/d, and the high dose was set at 60 mg/kg·bw/d.

#### Evaluating Indicators

Each treatment group was continuously administered with drug preparations and dosages for 7 days. After drug withdrawal, the animals were observed for 14 days. Weighing and temperature measurements were performed before and after administration. Feed consumption was recorded daily, and weight gain and feed conversion were calculated for each experimental group to evaluate the growth of animals in each group. Clinical symptoms were scored and monitored daily, with the detailed scores shown in Supplementary Table 1. During the trial, the morbidity, mortality, and cure rate of animals were observed and recorded to evaluate the therapeutic effect of drugs.

### Statistical Analysis

Experimental data were organized in MS Excel 2019. Results were expressed as mean and SEM (Standard Error of Mean). Repeated measures ANOVA were performed for data comparison using the SPSS 26.0 software (IBM^®^). In our analysis, when Mauchly's test of sphericity was *P* > 0.05, a within-subjects effect test was performed; otherwise, when *P* < 0.05, a multivariate test was performed. A simple effect analysis was performed if the interaction effects were significantly different. Bonferroni corrections were applied for *post-hoc* analysis. When *P* < 0.05, the difference was considered significant, and when *P* < 0.01, the difference was considered highly significant.

For 16S rRNA gene sequencing analysis, the original sequences were processed using Fastp software (Version 0.19.6, https://github.com/OpenGene/fastp) and spliced using FLASH software (version 1.2.11, https://ccb.jhu.edu/software/FLASH/index.shtml). The UPARSE software (version 7.0.1090, http://drive5.com/uparse/) was used to perform OTU clustering on the sequences, according to 97% similarity. Finally, the RDP classifier (Version 2.11, https://sourceforge.net/projects/rdp-classifier/) was used to classify the species of each sequence, comparing the Silva Database (SSU123) and using the alignment threshold of 70%.

After the quality control of the raw data, the OTU with 97% similarity was selected, and the alpha diversity index of samples was calculated using the Mothur software (Version 1.30.2 https://www.mothur.org/wiki/Download_mothur). Next, the R language drew the Rarefaction curve (Version 3.3.1 https://www.rproject.org/). Then, the beta diversity distance matrix was calculated using the Qiime software (Version 1.9.1 http://qiime.org/install/index.html), and the distance algorithm used was unweighted-unifrac. Then, the principal coordinates analysis (PcoA) diagram was analyzed using the R language, and the analysis of similarities (ANOSIM) was used to test the difference between groups. Finally, relative abundances at phylum and genus levels were calculated from the ratio of sequence numbers of species with total numbers.

For the therapeutic effect trial, the Chi-square (χ^2^) test was used to analyze the differences in the morbidity, mortality, and cure rates of the tested veterinary drug groups and the control groups. The degree of freedom (df) in this test was 1 when χ^2^ ≥ 3.84 indicated a significant difference; χ^2^ ≥ 6.63 indicated an extremely significant difference. In addition, one-way ANOVA with Duncan's multiple comparison test (SPSS 26.0 software) was used to analyze significant differences in the weight gain and feed conversion among groups.

## Results

### The Drug Preparation, Chlortetracycline Rumen-Protected Granules, Has Less Impact on Normal Rumen Microorganisms When Compared to the Premix

#### Copy Numbers of Eight Target Microorganisms in Different Drug Treatments

**Figure 1:**
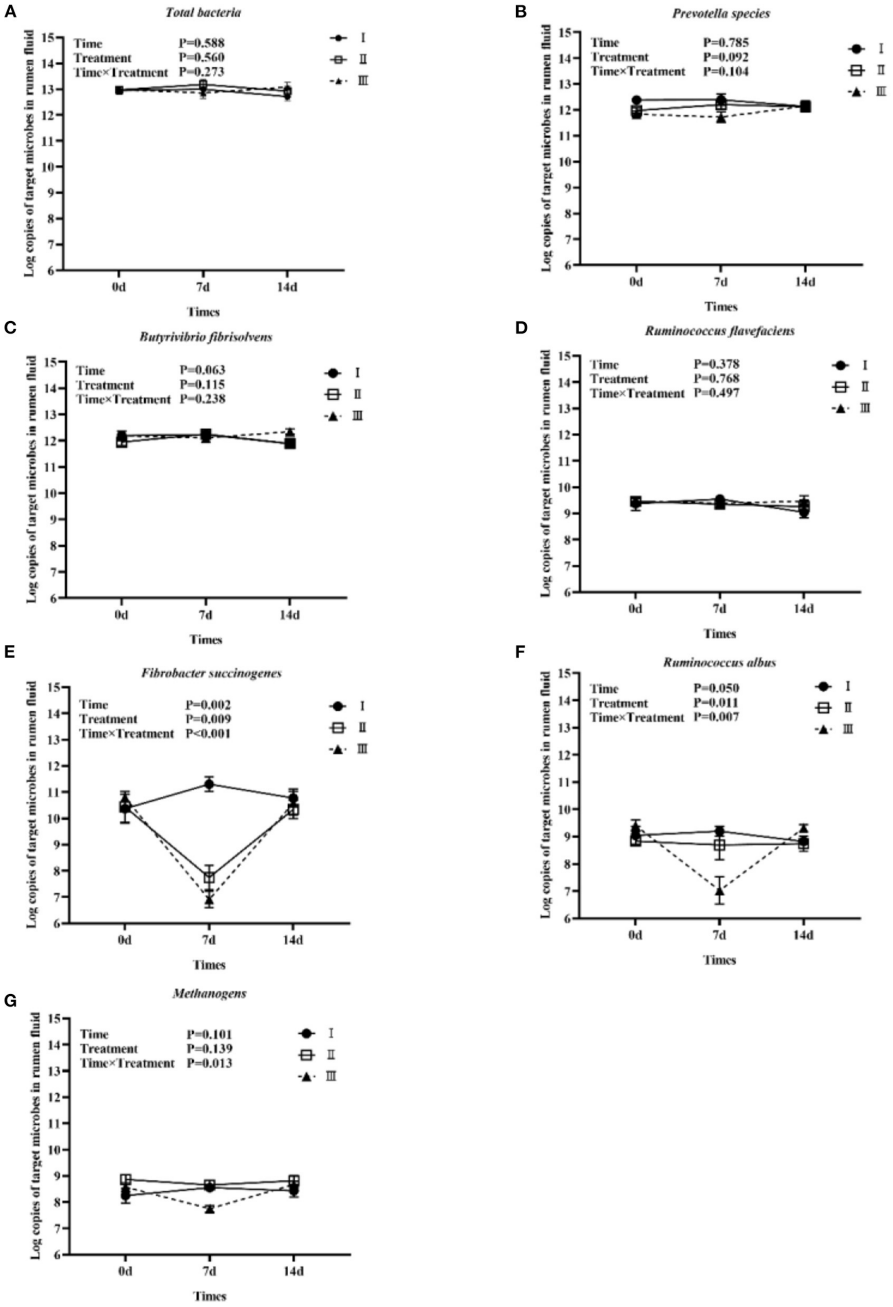
Trends of eight target microorganisms during the experiment. **(A–G)** represent the log copy numbers of *Total bacteria, Prevotella species, B. fibrisolvens, R. flavefaciens, F. succinogenes, R. albus*, and *Methanogens* during the 14-day trial, respectively. I was the blank control group; II was chlortetracycline rumen-protected granules treatment; III was chlortetracycline premix treatment.

Figure 1 shows the trends of eight target microorganisms in the rumen fluid of lambs. The copy numbers of *Total bacteria, Prevotella species, B. fibrisolvens*, and *R. flavefaciens* did not change significantly during the 14-day trial in all groups. Since the interaction effect of *F. succinogenes, R. albus*, and *Methanogens* were significantly different (*P* < 0.001, 0.007, and 0.013, respectively), simple effect analysis and multiple comparison were conducted. Results are shown in Tables 2, 3. With 7 days of granules and premix, the gene copy numbers for *F. succinogenes* decreased significantly, from 2.74 × 10^10^ copies/mL to 5.58 × 10^7^ copies/mL and 6.19 × 10^10^ to 8.13 × 10^6^ copies/mL, compared to the control. Seven days after treatment, the gene copy numbers in granules and premix groups recovered to 2.19 × 10^10^ copies/mL and 3.85 × 10^10^ copies/mL, respectively (Figure 1E). For *R. albus* and *Methanogens*, there were no significant differences in gene copy numbers between granules and control groups; however, the premix group showed significantly lower copy numbers than granules and control groups after 7 days of drug administration, resulting in recovery to pre-administration level after the treatment (Figures 1F,G). It is worth noting that in our trial, through qPCR assay, the Ct value of *General anaerobic fungi* was greater than 35 after 7 days of the administration, resulting in the non-detection of its copy numbers. Altogether, these results demonstrate that granules and premix can diminish the number of some rumen microorganisms compared with the control group. However, our self-developed granules showed a lesser effect on the copy number of rumen microorganisms than the premix.

**Table 2:** Log copies of a target microorganism in rumen fluid of lambs (Multiple comparisons between treatment at a specific time).

**Species**	**I**			**II**			**III**			**SEM**	**Treatment**	**Time**	**Interaction**
	**0 d**	**7 d**	**14 d**	**0 d**	**7 d**	**14 d**	**0 d**	**7 d**	**14 d**		***P*-value**	***P*-value**	***P*-value**
*Total bacteria*	12.95	13.00	12.71	12.97	13.18	12.92	12.98	12.86	13.09	0.055	0.560	0.588	0.273
*Prevotella species*	12.38	12.39	12.13	11.97	12.21	12.12	11.83	11.72	12.15	0.060	0.092	0.785	0.104
*B. fibrisolvens*	12.19	12.23	11.88	11.94	12.25	11.89	12.18	12.10	12.34	0.052	0.115	0.063	0.238
*R. flavefaciens*	9.37	9.55	9.04	9.46	9.35	9.26	9.46	9.39	9.47	0.063	0.768	0.378	0.497
*F.succinogenes*	10.37	11.30	10.78	10.44^a^	7.75^b^	10.34^a^	10.79^A^	6.91^B^	10.59^A^	0.232	0.009	0.002	<0.001
*R. albus*	9.04	9.20	8.82	8.83	8.69	8.74	9.41^Aab^	7.03^Bb^	9.31^ABa^	0.132	0.011	0.050	0.007
*Methanogens*	8.25	8.55	8.43	8.86	8.66	8.81	8.58^ABa^	7.75^Bb^	8.67^Aab^	0.072	0.139	0.101	0.013
*General anaerobic fungi*	8.00	8.34	7.82	8.31	N.D.	7.92	7.67	N.D.	8.06	0.463	–	–	–

*I was the infection control group; II was the chlortetracycline rumen-protected granules group; III was the chlortetracycline premix group. SEM means standard error of the mean (n = 6). N.D., none detected*.

*The letter denotes significant difference between the time at specific treatment. The difference was significant if there were no same small letters in the same line (P < 0.05), and the difference was very significant if there were no same capital letters (P < 0.01); There was no significant difference with the same letter (P > 0.05)*.

**Table 3:** Log copies of a target microorganism in rumen fluid of lambs (Multiple comparisons between time at a specific treatment).

**Species**	**0 d**			**7 d**			**14 d**			**SEM**	**Treatment**	**Time**	**Interaction**
	**I**	**II**	**III**	**I**	**II**	**III**	**I**	**II**	**III**		***P*-value**	***P*-value**	***P*-value**
*Total bacteria*	12.95	12.97	12.98	13.00	13.18	12.86	12.71	12.92	13.09	0.055	0.560	0.588	0.273
*Prevotella species*	12.38	11.97	11.83	12.39	12.21	11.72	12.13	12.12	12.15	0.060	0.092	0.785	0.104
*B. fibrisolvens*	12.19	11.94	12.18	12.23	12.25	12.10	11.88	11.89	12.34	0.052	0.115	0.063	0.238
*R. flavefaciens*	9.37	9.46	9.46	9.55	9.35	9.39	9.04	9.26	9.47	0.063	0.768	0.378	0.497
*F.succinogenes*	10.37	10.44	10.79	11.30^A^	7.75^B^	6.91^B^	10.78	10.34	10.59	0.232	0.009	0.002	<0.001
*R. albus*	9.04	8.83	9.41	9.20^A^	8.69^AB^	7.03^B^	8.82	8.74	9.31	0.132	0.011	0.050	0.007
*Methanogens*	8.25	8.86	8.58	8.55^a^	8.66^a^	7.75^b^	8.43	8.81	8.67	0.072	0.139	0.101	0.013
*General anaerobic fungi*	8.00	8.31	7.67	8.34	N.D.	N.D.	7.82	7.92	8.06	0.463	–	–	–

*I was the infection control group; II was the chlortetracycline rumen-protected granules group; III was the chlortetracycline premix group. SEM means standard error of the mean (n = 6). N.D., none detected*.

*The letter denotes significant difference between treatments at a specific time. The difference was significant if there were no same small letters in the same line (P < 0.05), and the difference was very significant if there were no same capital letters (P < 0.01); There was no significant difference with the same letter (P > 0.05)*.

#### 16S rRNA Gene Sequencing Analysis of Rumen Microorganisms in Different Drug Treatments

After sequencing the 16S rRNA gene amplicon of 54 samples (18 samples from the control group, 18 samples from the granules group, 18 samples from the premix group), a total of 6,304,790 raw reads were obtained. After further impurity removal and pruning, 3,152,395 optimized sequences were obtained with an average sequence length of 417 bp. In addition, 2,821 OTUs were obtained after clustering of sequences. Our samples showed a 97% homology with 26 phyla, 60 classes, 132 orders, 229 families, 447 genera, and 847 species. We used the Chao1 rarefaction curve to evaluate a sufficient sequencing quantity of samples. As shown in Supplementary Figure 4, the dilution curve of each sample gradually flattened with the increase of sequencing depth, indicating that the sequencing depth of this test was sufficient to cover the vast majority of microorganisms in the samples.

To further analyze the differences in species richness and diversity among the three groups, we measured the Shanno index, Chao 1 index, Ace index, and Simpson index of the samples. The interaction effects of the Chao 1 index and Ace index were significantly different (*P* = 0.014, and 0.023, respectively). Hence, a simple effect analysis was conducted in the analysis. Bonferroni corrections were applied for *post-hoc* multiple analysis. As shown in Tables 4, 5, compared to the control group, Chao1 index and Ace index decreased significantly after 7 days of continuous administration of both premix and granules (*P* < 0.05). For the Shanno index, since *P*-values from the two within-subject effects were significant and there were three levels for the two within-subject effects, Bonferroni corrections were applied for *post-hoc* multiple analysis of treatment and time main effects. Results showed that, for the main effect of treatment, the Shanno index of the premix group was significantly different from that of the control group (*P* = 0.001). However, there was no significant difference between the granules and control groups (*P* = 0.297). For the main effect of time, the Shanno index at 7 days after administration was significantly different than the pre-administration period (*P* = 0.044). For the Simpson index, the main effect of treatment showed a significant difference. After multiple comparisons, the results showed that the Simpson index of the premix group was significantly different from the control group (*P* = 0.020), but there was no significant difference between the granules group and control group (*P* = 0.662). These results demonstrate that granules and premix can diminish the richness and diversity of the microbial community.

**Table 4:** α diversity analysis results (Multiple comparisons between treatment at a specific time).

**Item**	**I**			**II**			**III**			**SEM**	**Treatment**	**Time**	**Interaction**
	**0 d**	**7 d**	**14 d**	**0 d**	**7 d**	**14 d**	**0 d**	**7 d**	**14 d**		***P*-value**	***P*-value**	***P*-value**
Shannon	3.68	3.70	3.79	3.81	3.04	3.44	3.62	3.04	3.23	0.059	0.037	0.035	0.094
Chao1	355.01	338.00	359.76	364.36^A^	265.61^B^	278.13^B^	372.35^Aa^	236.43^Ba^	310.94^ABb^	8.009	0.020	0.012	0.014
Ace	346.66	333.81	358.82	349.74^Aa^	252.46^Bab^	273.28^ABb^	365.15^Aa^	240.08^Ba^	311.36^ABb^	7.681	0.005	0.004	0.023
Simpson	0.07	0.05	0.07	0.05	0.12	0.08	0.07	0.12	0.12	0.007	0.025	0.193	0.092

*I was the infection control group; II was the chlortetracycline rumen-protected granules group; III was the chlortetracycline premix group*.

*SEM means standard error of the mean (n = 6)*.

*The letter denotes significant difference between the time at a specific treatment. The difference was significant if there were no same small letters in the same line (P < 0.05), and the difference was very significant if there were no same capital letters (P < 0.01); There was no significant difference with the same letter (P > 0.05)*.

**Table 5:** α diversity analysis results (Multiple comparisons between time at a specific treatment).

**Item**	**0 d**			**7 d**			**14 d**			**SEM**	**Treatment**	**Time**	**Interaction**
	**I**	**II**	**III**	**I**	**II**	**III**	**I**	**II**	**III**		***P*-value**	***P*-value**	***P*-value**
Shannon	3.68	3.81	3.62	3.70	3.04	3.04	3.79	3.44	3.23	0.059	0.037	0.035	0.094
Chao1	355.01	364.36	372.35	338.00^Aa^	265.61^ABb^	236.43^Bab^	359.76^Aa^	278.13^Bab^	310.94^ABb^	8.009	0.020	0.012	0.014
Ace	346.66	349.74	365.15	333.81^A^	252.46^B^	240.08^B^	358.82^Aa^	273.28^Bab^	311.36^ABb^	7.681	0.005	0.004	0.023
Simpson	0.07	0.05	0.07	0.05	0.12	0.12	0.07	0.08	0.12	0.007	0.025	0.193	0.092

*I was the infection control group; II was the chlortetracycline rumen-protected granules group; III was the chlortetracycline premix group*.

*SEM means standard error of the mean (n = 6)*.

*N.D., none detected*.

*The letter denotes significant difference between treatments at a specific time. The difference was significant if there were no same small letters in the same line (P < 0.05), and the difference was very significant if there were no same capital letters (P < 0.01); There was no significant difference with the same letter (P > 0.05)*.

**Figure 2:**
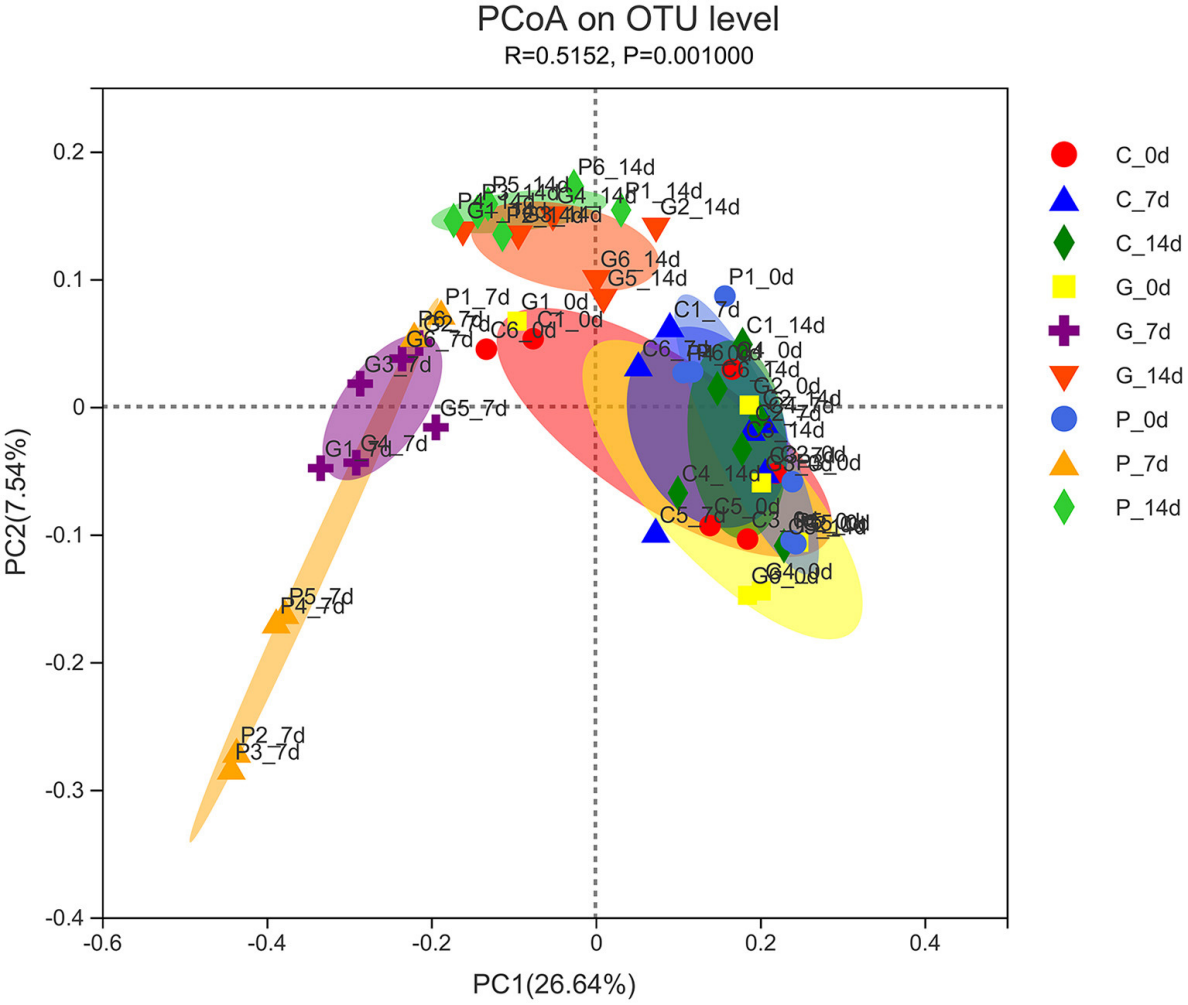
PCoA analysis of experimental groups. C_0 d, 7 d, 14 d represent the samples of the control group on 0 d, 7 d, 14 d, respectively; G_0 d, 7 d,14 d symbolize the samples of chlortetracycline rumen-protected granules group on 0 d, 7 d, 14 d, respectively; P_0 d, 7 d,14 d represent the samples of chlortetracycline premix group on 0 d, 7 d, 14 d, respectively. The horizontal and vertical axes represent the first and second principal coordinates explaining the most significant proportion of variance to the bacterial communities (showed by percentage). PCoA, principal coordinates analysis.

The PcoA analysis based on unweighted UniFrac showed that the microbiota structure significantly differed between the treatment groups (*P* = 0.001). As shown in Figure 2, the samples at day 0 of all experimental groups were close to each other and clustered in the same range, indicating that the initial rumen microbial community composition was similar. In the control group, samples from 0 to 14 days were clustered together, indicating a very similar composition of rumen microbiota. However, after 7 days of the administration, the ruminal microbiota communities in the granules and premix groups differed from those in the control group, especially the premix group, indicating that both granules and premix can affect the rumen microbiota and the premix group has a more significant impact than granules. After 7 days of drug withdrawal, the distance decreased significantly in both the treated group, suggesting that the composition of rumen bacteria gradually recovered after 7 days of drug withdrawal.

**Figure 3:**
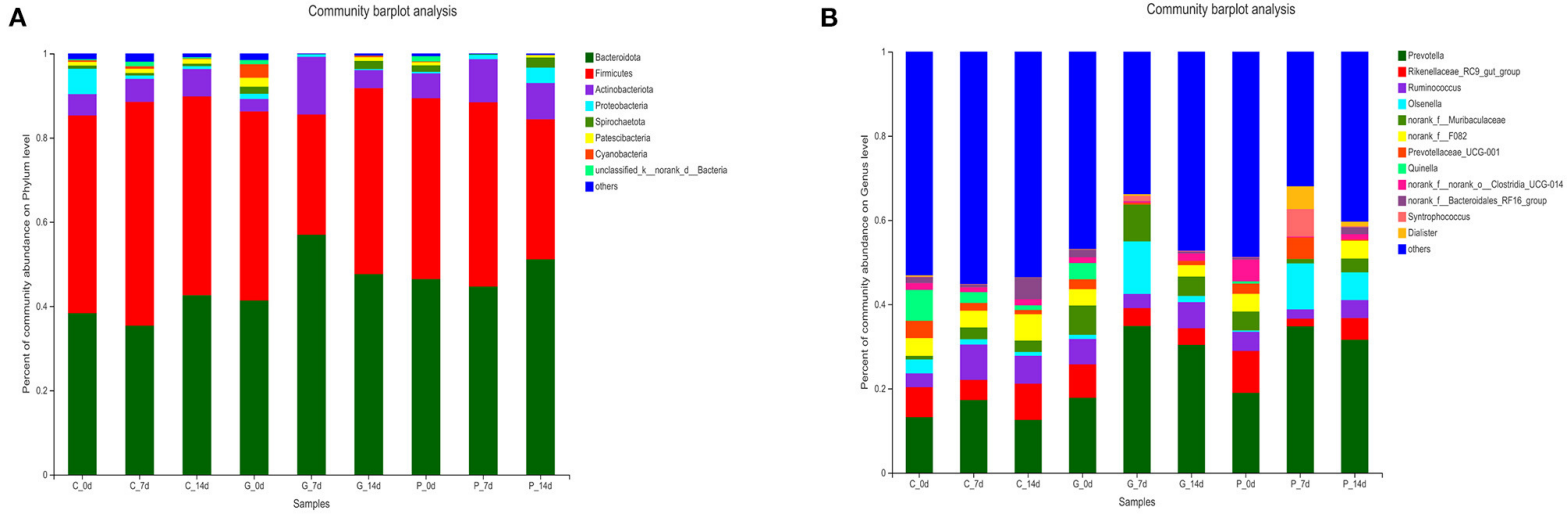
Composition of rumen bacterial community at the phylum level. **(A)** Bar chart of phylum level in each treatment group; **(B)** The proportion of each common bacterium at the phylum level.

To study further the effect of different treatments on rumen microbiota of lambs, we carried out a bar chart analysis at the phylum and genus level (Figures 3A,B). At the phylum level, as shown in Figure 3A, *Bacteroidetes* (35.38–56.94%) and *Firmicutes* (28.55–53.11%) were the dominant phyla in each treatment group, the total relative abundance was more than 80%. In addition, we found that rumen microbial species composition at the phylum level changed in granules and premix groups compared to the control group 7 days after administration. The relative abundance of *Actinnobacteriota* increased from 2.96 to 13.69% and 5.87 to 10.22%, respectively. Meanwhile, the relative abundance of *Spirochaetota, Patescibacteria* and *Cyanobacteria* decreased to 0%. At the genus level, 12 dominant bacterial genera were screened according to the relative abundance >5%, as shown in Figure 3B. The dominant genera in each group were *Prevotella* (12.53–34.76%), *Rikenellaceae_RC9_gut_group* (1.82–9.97%), and *Ruminococcus* (2.21–8.40%). After 7 days of the administration, rumen microbial species composition at the genus level changed in granules and premix groups. Compared with 0 d, both granules and premix increased the relative abundance of *Prevotella* (17.79 to 34.85% and 18.98 to 34.76%, respectively) and *Olsenella* (0.10 to 12.46% and 0.34 to 10.91%, respectively). The relative abundance of *norank_f_F082* decreased from 3.90% and 4.20 to 0%, respectively. In the granules group, the relative abundance of *Quinella* decreased from 3.83 to 0%. In the premix group, the relative abundance of *Prevotellaceae_UCG-001, Syntrophococcus* and *Dialister* increased from 2.41 to 5.09%, 0 to 6.53%, and 0 to 5.39%, respectively. These results indicate that feeding granules or premix affects the rumen microbiota composition of lambs, consistent with α diversity and PcoA analysis results.

### Therapeutic Effect Trial Shows That Chlortetracycline Rumen-Protected Granules Are Effective Against Lamb Diarrhea Caused by *E. coli*

**Figure 4:**
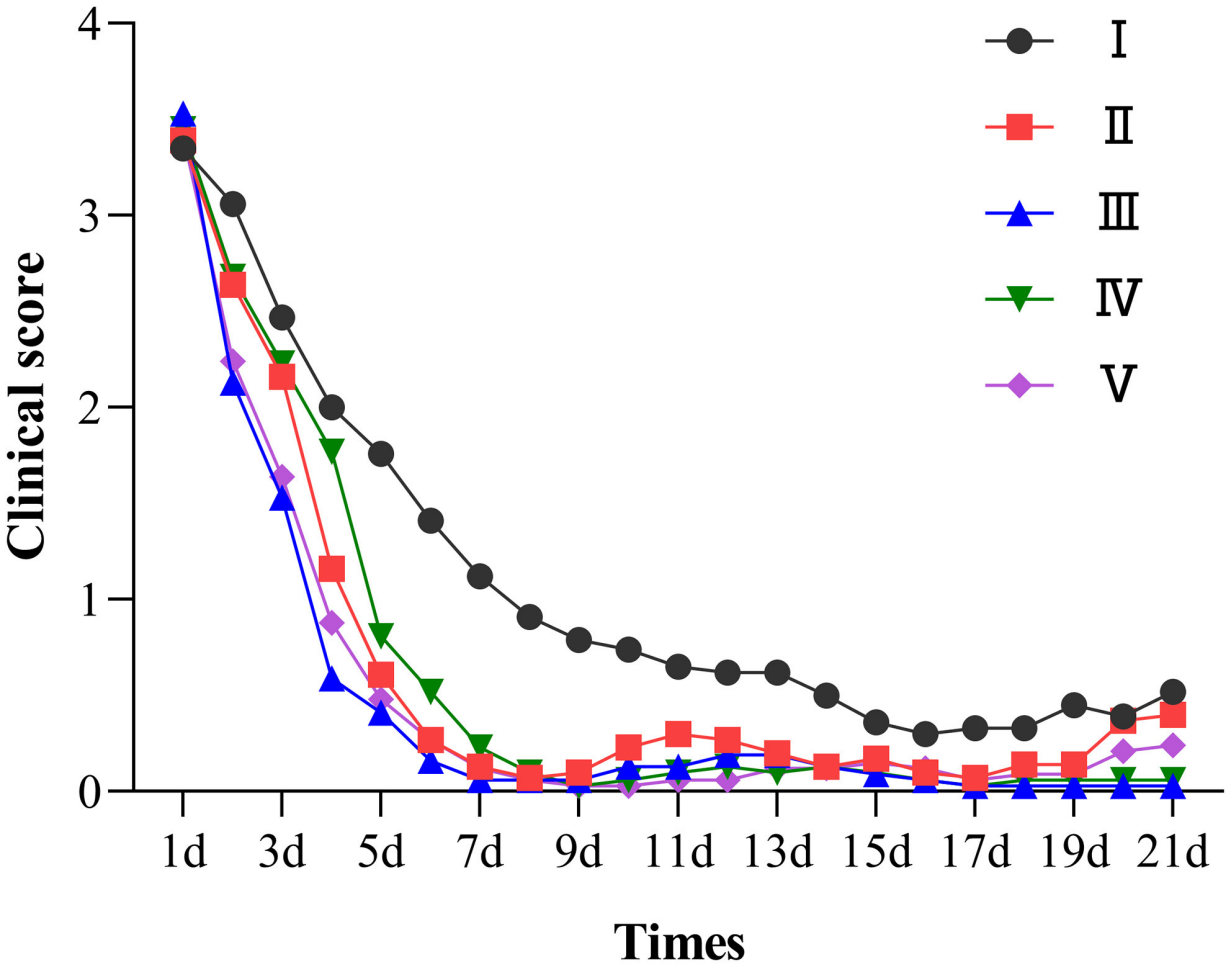
Clinical symptom score chart of each group. I was the infection control group; II, III, IV were the low, medium, and high dose groups of chlortetracycline rumen-protected granules, respectively; V was the chlortetracycline premix group; VI was the healthy control group.

A total of 195 clinical cases were collected in this study, and 161 cases were included for statistical analysis after pathogen identification. Figure 4 shows the observation and record of clinical progression in the test animals, daily score of clinical signs, and the trends after treatment. Before administration, the sick lambs showed listlessness, anorexia, and diarrhea. In addition, some animals with severe diarrhea showed weakness, depression, inappetence, rough and matte coat, trembling, unformed feces around the anus, and mushy feces. The specific clinical signs of sick lambs before drug administration are shown in Supplementary Figure 5. The healthy control group showed no clinical signs of diarrhea during the trial. In contrast, the treatment group exhibited a gradual improvement, showing less diarrhea and depression and a gradual return of appetite after 4 days of drug administration.

The cure rates of the chlortetracycline rumen-protected granules (medium-dose, high-dose, and low-dose), chlortetracycline premix, and infection control groups were 96.88, 93.55, 61.29, 75.76, and 52.94%, respectively (Table 6). We observed a significant difference in the cure rates among chlortetracycline rumen-protected granules (medium-dose and high-dose groups) and the infection control group (χ^2^ = 16.64 and 13.35, respectively). However, there was no significant difference in the cure rate among the chlortetracycline rumen-protected granules low-dose group, chlortetracycline premix group, and infection control group (χ^2^ = 0.46 and 3.79, respectively). During the trial, no death was found in each group.

**Table 6:** Clinical treatment effect of each group (with cure rate as evaluation index).

**Group**	**Number of animals**	**Effective rate%**	**Cure rate%**	**Death rate%**	**Compared with group I**	**Compared with group II**	**Compared with group III**	**Compared with group IV**
I	34	–	52.94^*^ (18/34)	0.00 (0/34)	–	–	–	–
II	31	100.00 (31/31)	61.29 (19/31)	0.00 (0/31)	χ^2^ = 0.46	–	–	–
III	32	100.00 (32/32)	96.88 (31/32)	0.00 (0/32)	χ^2^ = 16.64	χ^2^ = 12.18	–	–
IV	31	100.00 (31/31)	93.55 (29/31)	0.00 (0/31)	χ^2^ = 13.35	χ^2^ = 9.23	χ^2^ = 0.38	–
V	33	100.00 (33/33)	75.76 (25/33)	0.00 (0/33)	χ^2^ = 3.79	χ^2^ = 1.56	χ^2^ = 6.07	χ^2^ = 3.84
VI	39	–	–	–	–	–	–	–

*I was the infection control group; II, III, IV were the low, medium, and high dose groups of chlortetracycline rumen-protected granules, respectively; V was the chlortetracycline premix group; VI was the healthy control group*.

* *Stands for self-healing rate*.

*χ^2^chi-square test, when df = 1, χ^2^ ≥ 3.84 indicated significant difference; χ^2^ ≥ 6.63 indicated an extremely significant difference*.

After the experiment, we also observed a significant difference in average weight gain among the chlortetracycline rumen-protected granules medium-dose group, health control group, and infection control group (*P* < 0.01). Likewise, there was a significant difference between the chlortetracycline premix group and infection control group in terms of average weight gain (*P* < 0.05); however, there was no significant difference among the chlortetracycline rumen-protected granules medium-dose group, chlortetracycline premix group, and infection control group (*P* > 0.05) (Table 7).

**Table 7:** Changes of average body weight of lambs in each group.

**Groups**	**Initial weight (kg)**	**Final weight (kg)**	**Average weight gain (kg)**	**Average daily gain (kg)**
I	14.73 ± 2.11	15.52 ± 2.20	0.90^Bd^ ± 0.81	0.04^Bd^ ± 0.04
II	14.29 ± 2.16	15.32 ± 2.23	1.01^Bcd^ ± 0.78	0.05^Bcd^ ± 0.04
III	14.66 ± 2.17	16.30 ± 2.52	1.63^Aab^ ± 0.70	0.08^Aab^ ± 0.03
IV	13.86 ± 2.11	15.26 ± 2.42	1.40^ABbc^ ± 0.79	0.07^ABbc^ ± 0.04
V	15.23 ± 1.87	16.27 ± 2.00	1.04^Bcd^ ± 0.70	0.05^Bcd^ ± 0.03
VI	15.02 ± 1.89	16.85 ± 2.02	1.83^Aa^ ± 0.72	0.09^Aa^ ± 0.03

*I was the infection control group; II, III, IV were the low, medium, and high dose groups of chlortetracycline rumen-protected granules, respectively; V was the chlortetracycline premix group; VI was the healthy control group*.

*The difference was significant if there were no same small letters in the same column (P < 0.05), and the difference was very significant if there were no same capital letters (P < 0.01); There was no significant difference with the same letter (P > 0.05). One lamb was dissected in the group I and II, so the bodyweight at the end of the test did not include the bodyweight of dissected lamb*.

In conclusion, when the dose was increased from 15 to 30 mg/kg·bw/d, both the weight gain and the cure rate of the animals were significantly increased, but when the dose was increased to 60 mg/kg·bw/d, these evaluating indicators were decreased significantly. Similarly, in our daily observations, feed intake and feeding speed were significantly lower in the high-dose group than others. Therefore, the excessive dose may have adverse effects on animals. Compared with the premix group, the weight gain and feed conversion of the 15 mg/kg·bw/d granules group were approximately the same, and the cure rate was slightly lower but no significant difference. In comparison, the 30 mg/kg·bw/d was obviously better than the premix group in both weight gain and therapeutic effect.

**Figure 5:**
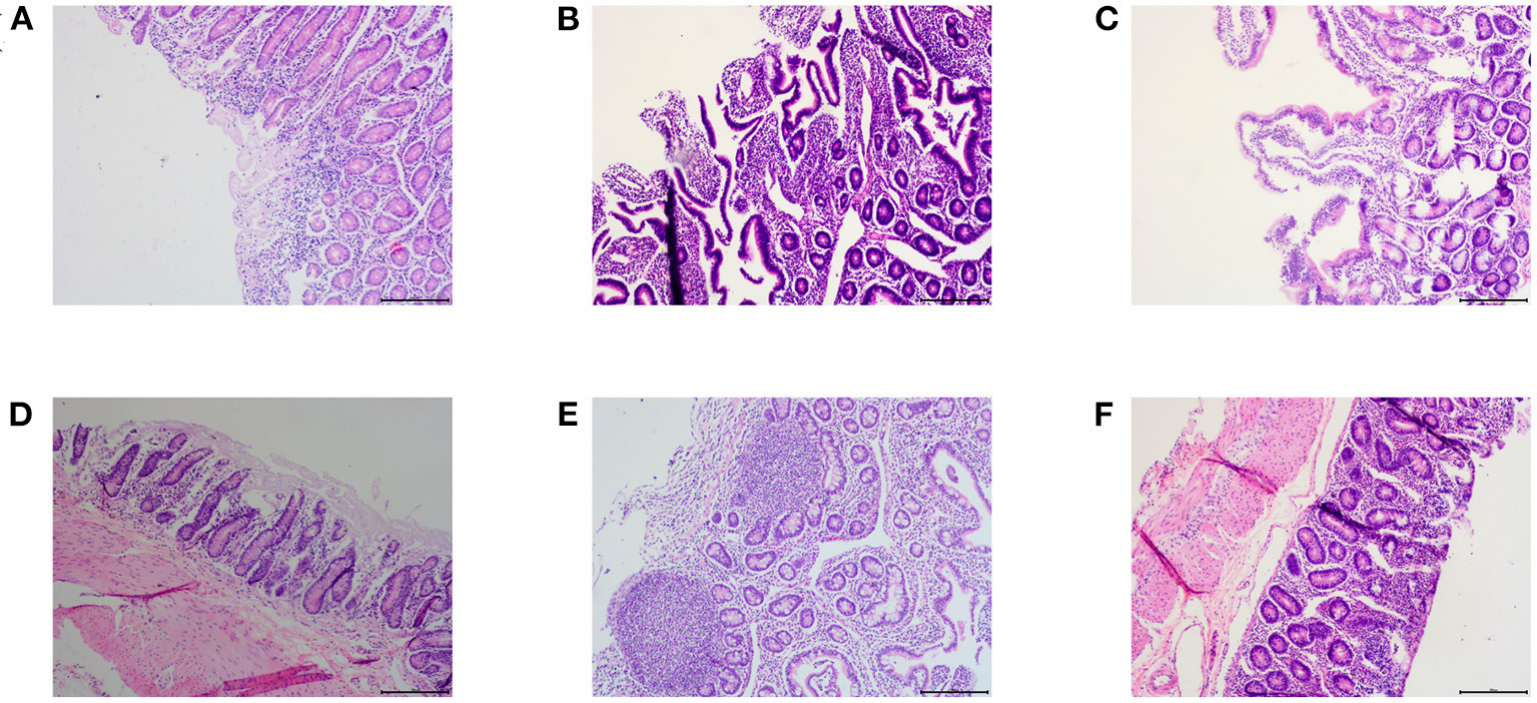
Pathological results (×100). **(A–C)** were the pathological results of the infected control group, **(A)** large infiltration of inflammatory cells in the duodenum; **(B)** Ileum mucosa was necrotic and exfoliated; **(C)** Jejunum mucosa was necrotic and exfoliated. **(D–F)** were the pathological results of the chlortetracycline rumen granules low-dose group, **(D)** no apparent abnormalities in the duodenum; **(E)** no apparent abnormalities in the ileum; **(F)** infiltration of eosinophils in the jejunum.

For a comprehensive comparison between treated and untreated animals 7 days after drug withdrawal, we examined the small intestine pathology (jejunum, ileum, and duodenum) of one sheep in the infection control group and the control chlortetracycline rumen granules low-dose group (Figure 5). The results of pathological sections showed that the jejunum, ileum, and duodenum of lambs in the control group were damaged after *E. coli* infection, showing exfoliation, necrosis, and infiltration of a large number of inflammatory cells. After drug administration, the intestinal injury was alleviated, although eosinophil infiltration remained in the jejunal mucosa. No apparent lesions were found in other tissues and organs, indicating that drug administration can effectively lessen the inflammatory reaction caused by *E. coli*.

## Discussion

The rumen development of lambs can be divided into three stages: non-ruminant, transitional, and rumination. Early study has shown that about 56 days after birth, the four stomachs of lambs have reached approximately an adult proportion (19). At this stage, rumen microbiota is stable, the dominant microbiota is prominent, and rumen starts to function. Therefore, we chose lambs about two and a half months old as the subject of this experiment. Our entire experiment lasted for 14 days, taking into account changes in the physiological state of animals in relation to the effect of the type of intervention given.

In our study, the population of all target microorganisms in the control group was maintained in a relatively stable range within 14 days, ensuring that the rumen was in a stable state. Therefore, the period difference had little effect on our experiment results at this stage. Furthermore, our data showed no significant changes in *Total bacteria* after treatment for two administration groups. Similarly, Klopfenstein et al. (20) came to the same conclusion that giving chlortetracycline to lambs did not significantly change the *Total bacteria* counts. However, it's worth noting that four of the eight target microorganisms in our study showed a significant decline in their population after the administration of chlortetracycline premix, and three of the four changes were related to cellulose digestion. Therefore, we conclude that chlortetracycline can inhibit ruminal cellulolytic bacteria, thus affecting cellulose digestion. This finding is consistent with Lodge et al. (21) results, in which chlortetracycline fed to cows or calves inhibited cellulose digestion by the rumen microorganism. Similarly, in 1953, scholars investigated that adding 1 g of chlortetracycline to the cattle diet may have deterred cellulose-digesting bacteria without affecting the total bacteria count (22). And in 1957, it was found that increasing chlortetracycline concentration decreased the digestibility of cellulose in feed (23). Only *F. succinogenes* and *General anaerobic fungi* changed significantly in the chlortetracycline rumen-protected granules. The range of change was smaller than premix, suggesting that the granules we developed can reduce the effect of chlortetracycline on rumen microorganisms to a certain extent compared to the chlortetracycline premix.

Many studies have shown that in cellulose digestion, the physical attachment of bacteria to the fiber material is essential (24, 25). Rumen pH is one of the factors affecting bacterial adhesion. Ruminal cellulolytic bacteria are susceptible to pH change. Researches have shown that the adhesion of *F. succinogenes* to cellulose was stable at pH 6–7, *R. flavefaciens* was stable at pH 3.3–7.5 (26) and *R. albus* was stable at pH 5.5–8 (27). Beacom et al. (28) found that chlortetracycline could reduce the pH of rumen fluid in cattle. Therefore, we suspect that chlortetracycline premix could inhibit ruminal cellulolytic bacteria by affecting the rumen pH, promoting the non-adhesion of bacteria to cellulose.

For *Methanogens*, previous studies have pointed out that bacteria and archaea have similar ribosomal structures, thus macrolides and tetracyclines can inhibit methanogenic archaea (29). Varel et al. (30) found that the addition of waste from cattle that had been fed chlortetracycline reduced the methane production rate by ~20%, in addition to interactions between rumen microorganisms. Morvan et al. (31) found that in the rumen digestive system of cattle, deer, sheep, and caecal samples from horses, *Methanogens* colonies were positively correlated with cellulose-digesting bacteria. In 1981, researchers confirmed that fungi could digest cellulose and found that fungi are closely associated with *Methanogens* (32). Thus, we suspect that the decline of *Methanogens* is due to the inhibitory effect of chlortetracycline on the one hand and the role of rumen cellulose bacteria and fungi on the other hand.

The 16S rRNA gene analysis confirmed that both premix and granules caused changes in rumen microbiota. However, in PCoA diversity analysis, granules had less influence on rumen microbiota than premixes, indicating that the damage to rumen microbiota caused by granules was less than that of the premix group. Many studies have shown that *Firmicutes* and *Bacteroidota* are the most dominant phyla in ruminants (33–36). Elie et al. discovered that *Firmicutes, Bacteroidota, Proteobacteria*, and *Actinobacteriota* had the highest relative abundance in rumen fluid, consistent with our sequencing results. In addition, *Fibrobacteres* were also detected in previous studies, but the abundance of *Fibrobacteres* was below 1% in our study. The samples collected in this study were rumen fluid. Previous studies have pointed out that *Fibrobacteres* were more common in the rumen solid phase than in the liquid phase (37), which may be the reason for the low relative abundance of phyla related to *Fibrobacteres* in our results.

At present, many studies have shown that chlortetracycline has a good prevention and treatment effect on clinical diarrhea. Puls et al. (38) found that feeding tiamulin in combination with chlortetracycline successfully controls respiratory and enteric diseases and, consequently, improves growth performance and carcass weight of grow-finish pigs. For ruminants, Murley et al. (39) found that after adding chlortetracycline to whole milk, the incidence of calf diarrhea decreased from 5.1 to 2.4%; after adding chlortetracycline to skimmed milk, the incidence of diarrhea decreased from 17.1 to 12.9%. Loosli and Wallace (40) found that adding crystalline chlortetracycline to the diet could significantly reduce the incidence and severity of diarrhea in calves. Similarly, our therapeutic effect trial shows that chlortetracycline can effectively improve lamb diarrhea, and compared with premix, the self-developed granules drug has a higher cure rate and has an excellent therapeutic effect on lamb *E. coli* diarrhea.

Therefore, based on all the trial results, we can conclude that compared with premix, our independently developed chlortetracycline rumen-protected granules can reduce the influence of the drug on rumen microbiota and have the effect of treating diarrhea. Thus, it provides a new way to reduce the impact of chlortetracycline on the rumen health of ruminants.

## Data Availability Statement

The original contributions presented in the study are included in the article/Supplementary Material, further inquiries can be directed to the corresponding author/s.

## Ethics Statement

The animal study was reviewed and approved by Institutional Review Board of South China Agricultural University (SCAU-IRB).

## Author Contributions

XH designed the experiment and supervised the entire study. YY, YX, and YS involved in feeding of the animal and sample collection. YY and XL participated in sample testing and data analysis. YY drafted and modified the manuscript. ZL was involved in the drawing of the figures. GL provided constructive suggestions on the experiment design. All authors contributed to the article and approved the submitted version.

## Funding

This study received funding from National Key Research and Development Program of China (2016YFD0501306).

## Conflict of Interest

The authors declare that the research was conducted in the absence of any commercial or financial relationships that could be construed as a potential conflict of interest.

## Publisher's Note

All claims expressed in this article are solely those of the authors and do not necessarily represent those of their affiliated organizations, or those of the publisher, the editors and the reviewers. Any product that may be evaluated in this article, or claim that may be made by its manufacturer, is not guaranteed or endorsed by the publisher.
