# Supplementary material for: Effects of Chlortetracycline Rumen-Protected Granules on Rumen Microorganisms and Its Diarrhea Therapeutic Effect
Source: Front Vet Sci. 2022 Feb 18;9:840442. doi: 10.3389/fvets.2022.840442 (PMC8894847; doi:10.3389/fvets.2022.840442)
Supplement: Supplementary file 1 [file Data_Sheet_1.docx]

Supplementary Material

# Supplementary Figures and Tables

## Detailed scoring sheet for phase II clinical trials

**Table S1.** Clinical symptom score

| Observation items | Judgment criteria | Score |
| --- | --- | --- |
| Fecal status | Normal, feces shaped, granular or semi granular, gray black, smooth surface | 0 |
|  | Soft, soft feces, shapeless, grayish white or grayish yellow | 1 |
|  | Ointment, the feces are mushy, gray white or grayish yellow, and the anus is polluted by feces | 2 |
|  | Watery, watery feces, grayish white or grayish yellow, anal skin turns red | 3 |
| Clinical symptoms | Normal spirit, normal food intake | 0 |
|  | Good mental state and reduced food intake | 1 |
|  | Depressed, significantly reduced food intake, slight bow | 2 |
|  | Mental depression, stop food intake, bow and waist uneasiness, bouncing and kicking, muscle tremor | 3 |

When the total score is 0, it is judged as normal; The total score is 1-2, which is mild diarrhea; The total score is 3-4, which is moderate diarrhea; The total score is 5-6, which is severe diarrhea.

## Rumen fluid description


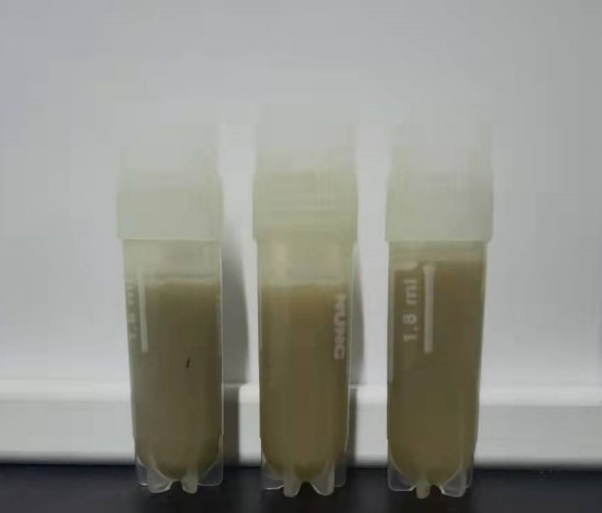


**Figure S1.** Rumen fluid samples. All rumen fluid samples were milky-brown and did not contain large particles.

## Rumen Bacteria DNA extraction

The OD value of the extracted DNA was between 1.8-2.0, indicating acceptable DNA purity. Furthermore, 1% gel electrophoresis showed that the target fragments were located at more than 2000 bp, with a clear and complete structure (Figure S2). All the results indicate that the extracted DNA can be used in further experiments.


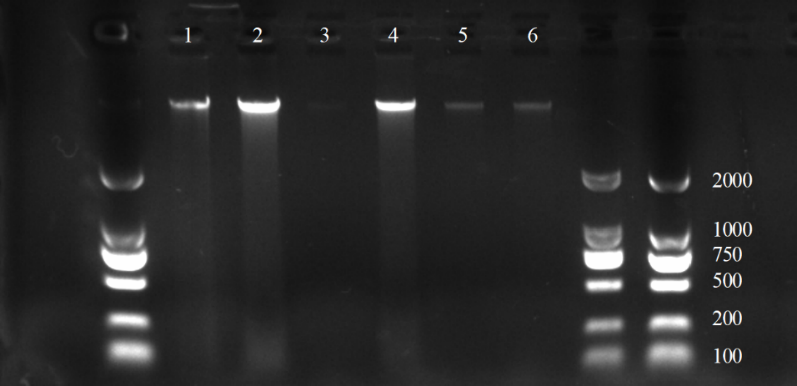


**Figure S2.** Agarose gel electrophoresis of total DNA in the rumen (part)

## Conventional PCR and Preparation of Standard Curve Plasmid

After conventional PCR amplification, agarose gel electrophoresis was performed on the PCR products of 8 species of rumen microorganism. Results showed that each target band was consistent with the predicted band, with no specific amplification band observed (Figure S3), indicating a reasonable specificity of the primers.

We sequenced the homology of the positive cloned plasmids through cloning and transformation in which all the homologies were above 95%, indicating that the plasmids containing the target fragment were successfully constructed.


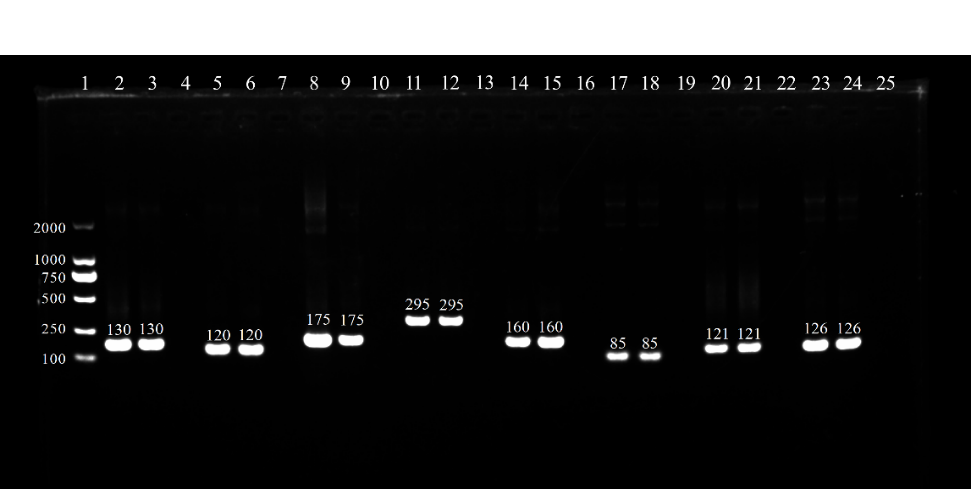


**Figure S3.** Agarose gel electrophoresis of rumen microorganism. 1:DL 2000 marker; 2-3: *General bacteria*(130bp); 5-6: *General anaerobic fungi*(120bp); 8-9: *Ruminococcus albus*(175bp); 11-12: *Ruminococcus flavefaciens*(295bp); 14-15: *Methanogens*(160bp); 17-18: *Prevotella species*(85bp); 20-21: *Fibrobacter succinogenes*(121bp); 23-24: *Butyrivibrio fibrisolvens*(126bp); 4,7,10,13,16,19,22,25: Negative control.

## RT-qPCR Standard Curve Efficiency

The standard curve equation of each bacterium is shown in Table S2. The R^2^ value of all standard curves was more than 0.99, and the amplification efficiency was between 90% - 110%.

**Table S2.** Standard curve equations

| Bacterial species | Standard curve equations | R-squared（R^2^） |
| --- | --- | --- |
| *General bacteria* | y=-3.294x+45.109  y=-3.349x+45.643  y=-3.282x+44.656 | 0.996  0.998  0.996 |
| *General anaerobic fungi* | y=-3.586x+44.544  y=-3.585x+44.275  y=-3.586x+44.105 | 0.995  0.994  0.992 |
| *Fibrobacter succinogenes* | y=-3.560x+47.346  y=-3.583x+46.822  y=-3.555x+47.223 | 0.993  0.995  0.994 |
| *Ruminococcus flavefaciens* | y=-3.469x+45.504  y=-3.469x+45.540  y=-3.506x+46.016 | 0.995  0.995  0.995 |
| *Ruminococcus albus* | y=-3.373x+43.826  y=-3.419x+44.101  y=-3.408x+43.401 | 0.997  0.996  0.998 |
| *Prevotella species* | y=-3.492x+46.524  y=-3.416x+44.084 | 0.998  0.993 |
| *Butyrivibrio fibrisolven* | y=-3.460x+47.505  y=-3.426x+47.332  y=-3.408x+43.722 | 0.995  0.996  0.994 |
| *Methanogens* | y=-3.211x+41.398  y=-3.317x+43.630  y=-3.303x+43.176 | 0.995  0.994  0.998 |

## Chao1 rarefaction curve


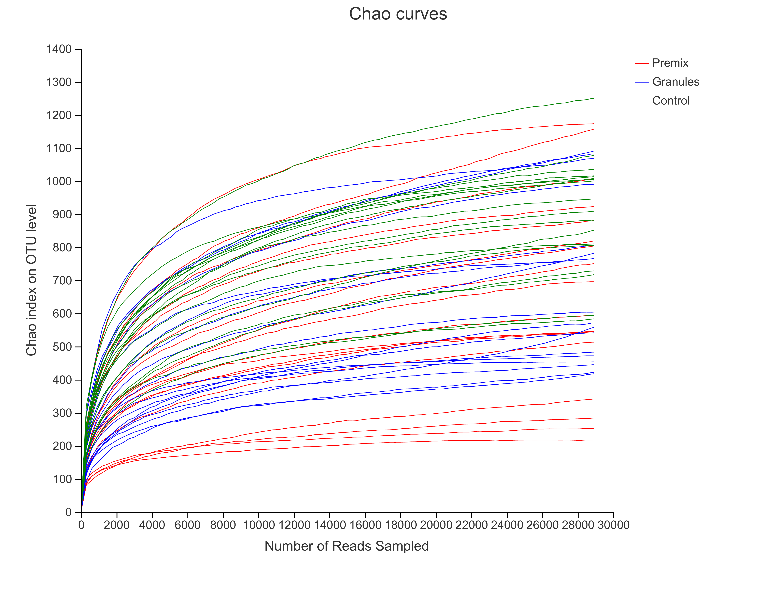


**Figure S4.** Chao1 rarefaction curve

## Pre-treatment status of phase II clinical cases


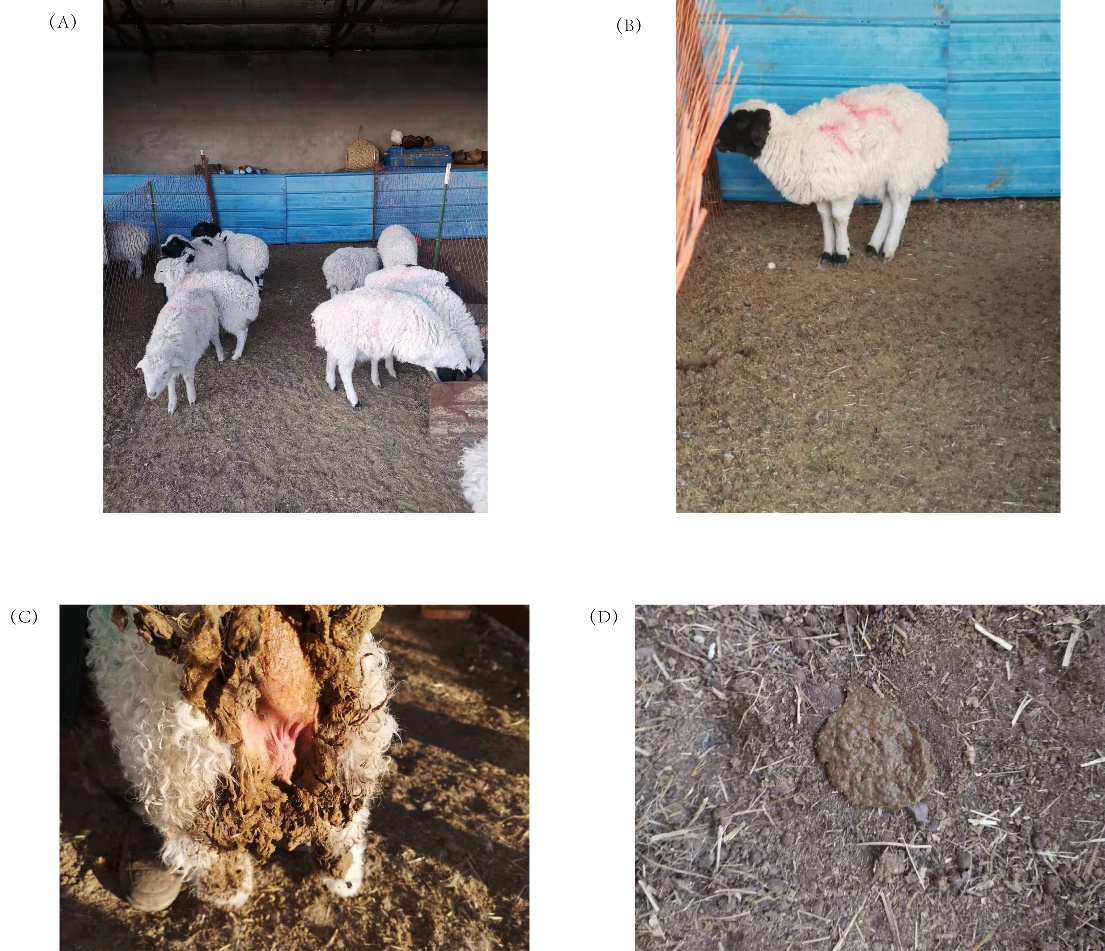


**Figure S5.** Clinical symptoms of the cases before treatment. (A) picture of inactive feeding; (B) Picture of a sick lamb hunching its back; (C) Picture of anal fecal contamination; (D) Picture of fecal character.
